# ASCT2 and LAT1 Contribution to the Hallmarks of Cancer: From a Molecular Perspective to Clinical Translation

**DOI:** 10.3390/cancers13020203

**Published:** 2021-01-08

**Authors:** Catarina Lopes, Carina Pereira, Rui Medeiros

**Affiliations:** 1 Molecular Oncology and Viral Pathology Group, IPO Porto Research Center (CI-IPOP), Portuguese Oncology Institute of Porto (IPO-Porto), Rua Dr. António Bernardino de Almeida, 4200-072 Porto, Portugal; catarina.p.lopes@ipoporto.min-saude.pt (C.L.); ruimedei@ipoporto.min-saude.pt (R.M.); 2 CINTESIS—Center for Health Technology and Services Research, University of Porto, Rua Dr. Plácido da Costa, 4200-450 Porto, Portugal; 3 Research Department of the Portuguese League Against Cancer—North (LPCC-NRNorte), Estrada da Circunvalação, 4200-177 Porto, Portugal

**Keywords:** alanine, serine, cysteine transporter 2, L-type amino acid transporter 1, hallmarks of cancer, clinical significance

## Abstract

**Simple Summary:**

ASCT2 and LAT1 are amino acid transporters whose impact in cancer has been explored throughout the years. They have been associated with most currently accepted hallmarks of cancer, thus the aim of this review is to report the impact of these transporters in this disease, as well as their clinical significance and applications. ASCT2 and LAT1 have been identified as prognostic factors and potentially as therapeutic targets. In conclusion, the study and development of new inhibitors for these amino acid transporters constitutes a promising approach towards the improvement of cancer treatment and prognosis.

**Abstract:**

The role of the amino acid transporters ASCT2 and LAT1 in cancer has been explored throughout the years. In this review, we report their impact on the hallmarks of cancer, as well as their clinical significance. Overall, both proteins have been associated with cell death resistance through dysregulation of caspases and sustainment of proliferative signaling through mTOR activation. Furthermore, ASCT2 appears to play an important role in cellular energetics regulation, whereas LAT1 expression is associated with angiogenesis and invasion and metastasis activation. The molecular impact of these proteins on the hallmarks of cancer translates into various clinical applications and both transporters have been identified as prognostic factors in many types of cancer. Concerning their role as therapeutic targets, efforts have been undertaken to synthesize competitive or irreversible ASCT2 and LAT1 inhibitors. However, JHP203, a selective inhibitor of the latter, is, to the best of our knowledge, the only compound included in a Phase 1 clinical trial. In conclusion, considering the usefulness of ASCT2 and LAT1 in a variety of cancer-related pathways and cancer therapy/diagnosis, the development and testing of novel inhibitors for these transporters that could be evaluated in clinical trials represents a promising approach to cancer prognosis improvement.

## 1. Introduction

Protein synthesis is essential to fuel the metabolic needs of cancer cell growth and relies on the maintenance of a homeostatic concentration of cytosolic amino acids, the primary source of cellular nitrogen [1,2,3]. Protein and amino acid synthesis and degradation, as well as amino acid uptake by transporters, are essential homeostasis regulators [1]. The latter are also important in cellular signaling and growth regulation, therefore highlighting potential targets for cancer therapy [3,4]. 

During the development of cancer and other metabolic diseases, proteins that confer growth and survival advantages, like amino acid transporters, are often overexpressed [3]. These transporters can be found in the plasma membrane or intracellular compartments such as the mitochondria, late endos40omes, lysosomes, and the Golgi apparatus [5]. Their dysregulation affects many functional factors, from intracellular energy metabolism to neurotransmission, and leads to metabolic reprogramming, triggering the carcinogenic process [5]. The neutral amino acid transporters solute carrier family A1 member 5/alanine, serine, cysteine transporter 2 (SLC1A5/ASCT2) and solute carrier family A7 member 5/L-type amino acid transporter 1 (SLC7A5/LAT1) ensure the rapid exchange of different amino acids and the maintenance of an amino acid pool in the cytosol, being likely involved in the development of several malignancies [4,5].

The hallmarks of cancer represent biological capabilities cancer cells acquire during the development of a tumor and help understand the vast diversity of neoplastic diseases [6]. This review will cover the function of ASCT2 and LAT1 transporters, their involvement in cancer, and their association with the hallmarks of cancer. Finally, it will address the clinical significance of both transporters, including their prognostic value and implication in therapy. 

### 1.1. Amino Acid Transport Systems: Focus on Systems ASC and L

In cells, amino acid transport across the plasma membrane is mediated by distinct transport systems [3]. Based on their substrate specificity, regulatory properties, and transport mechanism, amino acid transporters can be classified as sodium (Na^+^)-dependent neutral amino acid transporters, including systems A, ASC, where ASCT2 is included, B, and N; Na^+^-independent neutral amino acid transporters, gathering system L, that includes LAT1, and system T; Na^+^-dependent and -independent anionic amino acid transporters; and Na^+^-dependent and -independent cationic amino acid transporters [5,7]. Additionally, based on their sequence homology, these transporters can be included in different family groups [3]. Indeed, system ASC proteins belong to the solute carrier family 1 (SLC1) [3]. On the other hand, system L proteins are divided between two families, with LAT1 and LAT2 belonging to the SLC7 family, and LAT3 and LAT4 belonging to the SLC43 family [3]. 

As previously mentioned, system ASC includes Na^+^-dependent antiporters, ASCT1 and ASCT2, and it was originally named due to its affinity for the alanine, serine, and cysteine amino acids to differentiate it from system A [3,8]. This sodium ion dependency gives this system access to the energy stores inherent to the gradients of inorganic ions, making it able to generate gradients of amino acids in favor of the cytoplasmic environment [9]. 

System L activity is attributed to four Na^+^-independent transporters, LAT1, LAT2, LAT3, and LAT4 [3]. LAT1 and LAT2 have been shown to require the formation of covalent disulfide bridges between their 12 putative membrane-spanning domains and a type-II membrane glycoprotein heavy chain named 4F2hc to form a functional heterodimeric transporter [3]. On the contrary, LAT3 and LAT4 have been shown to not need 4F2hc to be functional [3]. These transporters mediate the uptake of bulky hydrophobic amino acids and are sensible to 2-amino-bicyclo[2.2.1]heptane-2-carboxylic acid (BCH), a synthetic competitive inhibitor [3].

#### 1.1.1. ASCT2: Function and Structure

**Figure 1:**
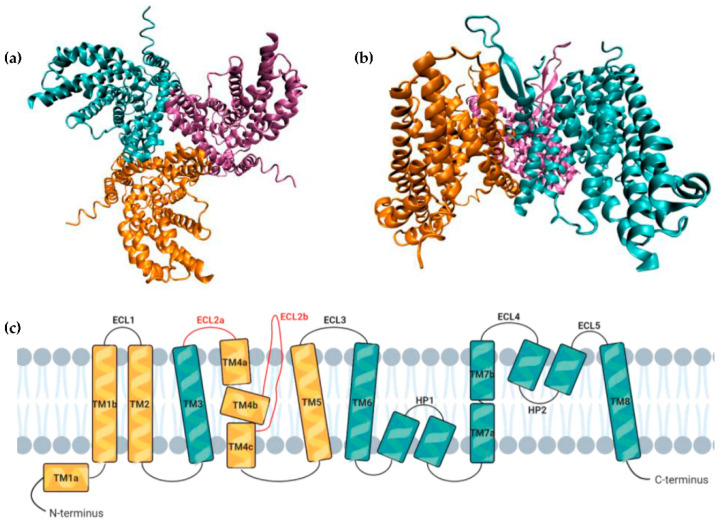
ASCT2 structure. (**a**) Bottom view highlighting the three protomers that constitute the ASCT2 homotrimer (PDB 6GCT); (**b**) Side view of the homotrimer (PDB 6GCT); (**c**) Membrane topology of a ASCT2 protomer with the scaffold domain in yellow and the transport domain in blue. The ECL2, that connects the two domains, is represented in red (adapted from [11,16] and created with BioRender.com). ECL: extracellular loop, HP: helical loop, TM: transmembrane segment.

ASCT2 is encoded by the *SLC1A5* gene and its structure shows a homotrimer (Figure 1a,b) [10,11]. It functions as an amino acid exchanger (antiporter) and accepts only neutral amino acids for transport in both directions [12]. Each ASCT2 protomer consists of a transport and a scaffold domain connected by the extracellular loop region 2 (ECL2a and ECL2b) [11]. The former contains the transmembrane segments (TMs) TM3, TM6-TM8 and the helical loops (HP) HP1 and HP2, whereas the latter includes the helices TM1, TM2, TM4, and TM5 [11]. Each transport domain interacts only with the scaffold domain from its own protomer and the ECL2 bridges the two domains, connecting TM3 to TM5 via TM4 (Figure 1c) [11]. There is a different number of Cys residues among SLC1 family members, with the only one conserved corresponding to C363 in ASCT2 [13]. Moreover, the reduction (SH) or oxidation (S-S) of Cys residues allows the switch of the protein state between activated and inactivated [13]. Unlike transporters from other systems, Na^+^ to K^+^ or L^+^ substitution is not tolerated and, contrary to LAT1, it is not inhibited by BCH [8,14]. The mechanism through which this transporter operates remains unclear, nevertheless, it is known that the exchange of amino acids is electroneutral and involves the cotransport of one Na^+^ ion and one neutral amino acid into the cytosol coupled with the cotransport of also one Na^+^ ion and one neutral amino acid out of the cell [3].

The ASCT2 transporter shows an asymmetric specificity, with alanine, methionine, and valine being transported only into the intracellular environment and asparagine, glutamate, serine, and threonine being carried in both directions [8]. Cysteine has been shown to work not as a ASCT2 substrate, but as a modulator at slightly higher concentrations, since it triggers efflux by uniport mode [15]. Glutamine is a preferred substrate and it has been shown that its uptake in several cancer cell lines is mediated mainly by this transporter [12].

#### 1.1.2. LAT1: Function and Structure

**Figure 2:**
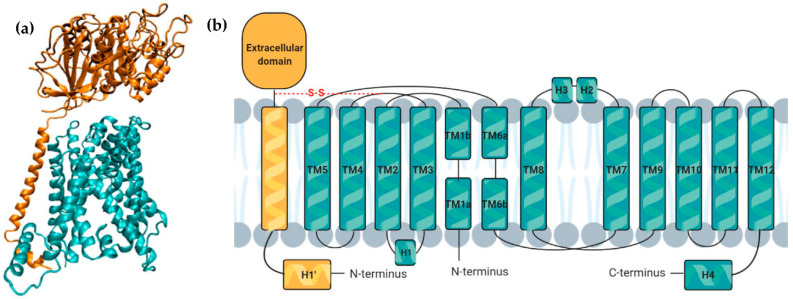
LAT1 structure. (**a**) Representation of the LAT1-4F2hc complex, with 4F2hc in orange and LAT1 in blue (PDB 6IRT); (**b**) Membrane topology of LAT1 transporter with the 4F2hc in yellow and the functional light chain LAT1 in blue. The disulfide bridge (S-S), that connects the two chains, is represented in red (adapted from [25] and created with BioRender.com). H: short helix, TM: transmembrane domain.

LAT1 is a heterodimeric amino acid transporter (HAT) that functions as an Na^+^-independent antiporter [17]. It consists of a functional light chain, LAT1, a 55 kDa protein encoded by the *SLC7A5* gene, that links through a conserved disulfide bridge to a heavy chain (4F2hc, also known as CD98), a 68 kDa type II glycoprotein encoded by the *SLC3A2* gene, that functions as a chaperone to recruit the functional subunit to the plasma membrane, stabilizing it (Figure 2a) [12,17,18,19,20,21,22,23]. LAT1 consists of 12 TMs, with TM1, TM3, TM6, TM8, and TM10 comprising the inner layer, which is surrounded by TM2, TM4, TM5, TM7, TM9, TM11, and TM12, the outer layer [24]. On the other hand, 4F2hc only comprises a single TM [24]. The complex also contains several loop and helical segments (H1–H4) [25]. The disulfide bridge forms between two conserved cysteine residues, one in the loop between transmembrane helices 3 and 4 of LAT1 and the other in 4F2hc (Figure 2b) [17]. LAT1 belongs to the *SLC7* gene family, is not glycosylated, and exhibits an intracellular N- and C-terminus, whereas the latter belongs to the *SLC3* gene family, is *N*-glycosylated, and presents an intracellular N-terminal and a bulky extracellular C-terminal [8,12]. Its substate-binding site consists of a proximal pocket that accommodates primary side chains, a distal pocket that functions as a binding site for hydrophobic secondary substitutions, and the positive and negative poles of two short helices that recognize the carboxyl and amino groups of the substrates [23]. LAT1 has a high affinity for bulky branched-chain and aromatic amino acids, particularly leucine, phenylalanine, and tryptophan, and can transport both D- and L-amino acid enantiomers [3,12,22,26]. Alanine, proline, and charged amino acids are not recognized by this transporter and it has lower affinity for glutamine and threonine [8]. As an obligatory amino acid exchanger with a 1:1 stoichiometry and lower affinity for substrates located within the cell its velocity is dependent on the concentration of the intracellular amino acids [3]. LAT1 has been shown to transport several drugs across the blood-brain barrier (BBB), being expressed at 100-fold higher levels in BBB endothelial cells than in other tissues [22].

### 1.2. ASCT2 and LAT1: Their Expression in Cancer

In a review by Fuchs et al. [3], ASCT2, LAT1, and sodium-coupled amino acid transporter 5 (SNAT5) expression stood out as significantly enhanced in tumors compared to their normal counterparts. However, whereas the latter had not been verified in other studies, ASCT2 and LAT1 expression pattern was similar in a variety of cancerous tissues (Table 1) [3].

Comparing to LAT1, ASCT2 transports a much wider range of substrates, namely glutamine as previously mentioned [3]. In fact, increased expression of this transporter has been documented in tissues where glutamine plays a particularly important role in metabolism, such as the liver, brain and the epithelial cells of the gut [3]. Moreover, enhanced ASCT2 expression has also been reported in cancers derived from tissues where this protein is not usually present [14].

Upregulation of both LAT1 and 4F2hc has been reported in cancer tissues or proliferative cells [12,27]. LAT1 has been detected in RERF-LC-MA lung small cell carcinoma cells, leukemia cell lines, T24 bladder carcinoma cells, and HeLa uterine cervical carcinoma cells and its expression has been correlated with the size of metastatic distant tumors in rats, thus being suggested as a potential therapeutic target for many cancers [3].

## 2. ASCT2 and LAT1: Their Contribution to the Hallmarks of Cancer

The hallmarks of cancer have been proposed by Hanahan and Weinberg and consist of biological concepts that allow the characterization of the heterogeneous and complex nature of cancer [6]. They consist of six capabilities that promote tumor progression (resisting cell death, sustaining proliferative signaling, activating invasion and metastasis, inducing angiogenesis, enabling replicative immortality, and evading growth suppressors) and are accompanied by two enabling characteristics (tumor-promoting inflammation and genomic instability and mutation) and two emerging hallmarks (avoiding immune destruction and dysregulating cellular energetics) that represent the important aspect of the constitution and signaling of the tumor microenvironment [6,66].

**Figure 3:**
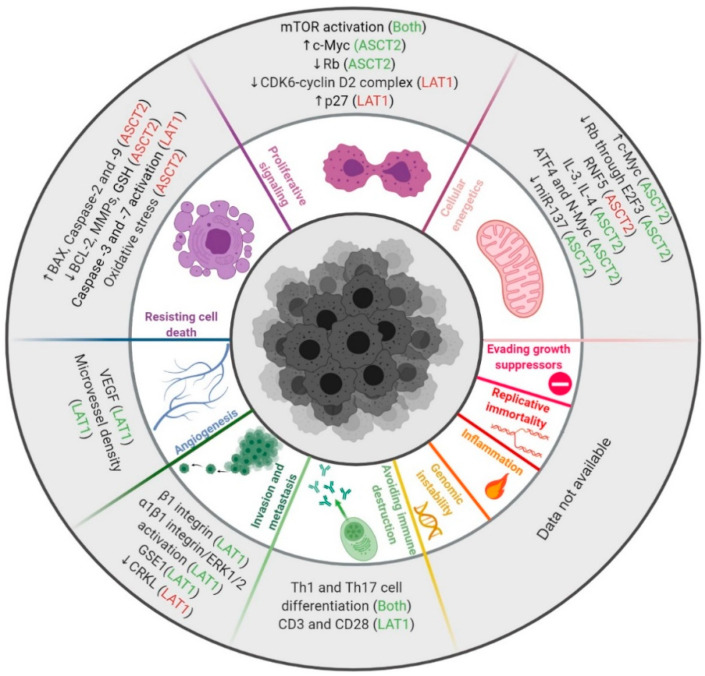
ASCT2, LAT1, and the hallmarks of cancer. Both ASCT2 and LAT1 have been associated with avoiding immune destruction [7,67,68,69,70,71,72], invasion and metastasis [47,73,74,75], angiogenesis [63,74,76,77,78], resisting cell death [20,29,79,80,81,82,83,84,85,86,87], proliferative signaling [43,52,87,88,89,90,91,92,93,94,95,96,97,98,99], and cellular energetics [90,100,101,102,103,104,105,106,107,108]. The activation/overexpression (green) or inactivation/subexpression (red) of those molecules consequently results in the dysregulation of a variety of key players on those hallmarks of cancer, culminating in cancer. The role of ASCT2 and LAT1 in genomic instability and mutation, inflammation, replicative immortality, and evading growth suppressors remains to be explored (created with BioRender.com).

The role of the amino acid transporters ASCT2 and LAT1 has not yet been explored in the context of two of the six original hallmarks, enabling replicative immortality and evading growth suppressors, nor of the enabling characteristics. In this section, we will explore the association between ASCT2 and LAT1 and these distinctive and complementary hallmarks of cancer (Figure 3).

### 2.1. Resisting Cell Death

Evasion of programmed cell death or apoptosis represents a major way of increasing the rate of cell proliferation during tumor progression [66]. Overall, apoptosis can result from an intrinsic or extrinsic pathway and both culminate in the activation of proteases named caspases [109]. The former, also known as the mitochondrial pathway, is mainly regulated by the B-cell lymphoma 2 (BCL-2) family of proteins, which can be activated by a variety of cellular stress signals, such as hypoxia, DNA damage and nutrient deprivation, and includes the anti-apoptotic proteins BCL-2 and BCL-x_L_ and the pro-apoptotic effectors BCL-2-associated X protein (BAX) and BCL-2 antagonist/killer (BAK), among others [109]. The extracellular pathway, on the other hand, can result from the binding of specific ligands to death receptors from the tumor necrosis factor (TNF) superfamily located in the cell surface, activating them [109]. Regarding the caspases involved in programmed cell death, caspase-2, -8, -9, and -10 are considered apoptosis initiators, whereas caspase-3, -6, and -7 are apoptosis executioners or effectors [110].

**Figure 4:**
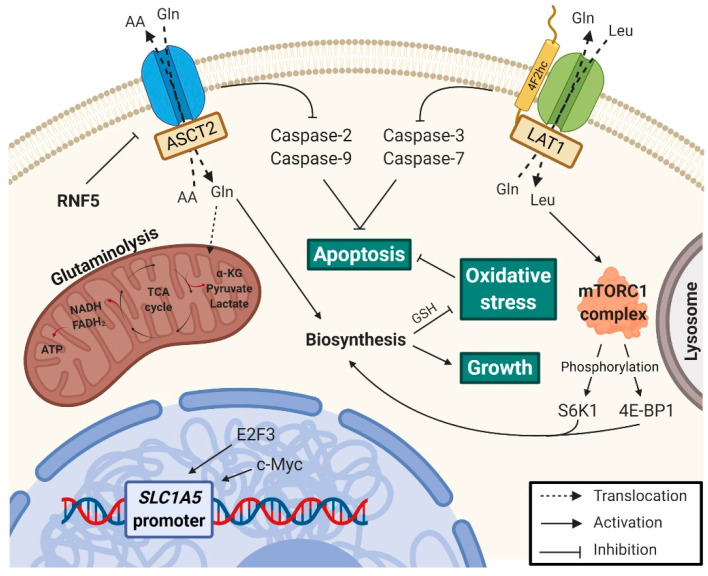
Pathways affected by ASCT2 and LAT1. ASCT2 is responsible for the transport of neutral amino acids into the cell, namely glutamine (Gln), coupled with the transport of one neutral amino acid (AA) to the extracellular environment. Gln can be used to synthesize glutathione (GSH), an antioxidant which prevents oxidative stress. In turn, together with ASCT2-related downregulation of caspases-2 and -9 and LAT1-related downregulation of caspases-3 and -7, oxidative stress inhibits apoptosis. On the other hand, Gln can also be used to biosynthesize other elements that play key roles in cell growth or be transported to the mitochondria and enter the glutaminolysis process, yielding metabolites, such as α-KG, pyruvate and lactate, and generating NADH and FADH_2_, essential for ATP production. Similar to ASCT2, LAT1 is an amino acid exchanger, transporting one amino acid into the cytosol, like leucine (Leu), and another amino acid to the extracellular milieu, namely Gln. Leu is required for the activation of the mammalian target of rapamycin complex (mTORC1), which in turn activates the downstream effectors ribosomal protein S6 kinase 1 (S6K1) and eukaryotic translation initiation factor 4E-binding protein 1 (4E-BP1) through phosphorylation. Those proteins play important roles in mRNA and protein biosynthesis, resulting in cell growth. In the nucleus, proto-oncogenes and tumor suppressors, such as c-Myc and E2F transcription factor 3 (E2F3), respectively, have been found to bind to promoter elements of the gene encoding ASCT2, resulting in altered Gln metabolism through *SLC1A5* dysregulation (created with BioRender.com).

ASCT2 knockdown has shown to play a role in the mitochondrial pathway of apoptosis, as it has been associated with decreased BCL-2 levels, increased BAX expression and a loss of matrix metallopeptidases (MMPs), which results from the mitochondrial membrane permeabilization facilitated by BAX [79]. Wang et al. [79], using a reactive oxygen species (ROS) scavenger, NAC, reported a reversion of the effects of ASCT2 knockdown in gastric cancer cell lines, supporting that this protein ablation leads to oxidative stress and contributes to apoptosis. That involvement was also observed in colorectal cancer cell lines using a synthetic small interfering RNA (siRNA) to attenuate the expression of *SLC1A5* [29] and in non-small cell lung cancer (NSCLC) using the l-γ-glutamyl-*p*-nitroanilide (GPNA) ASCT2 inhibitor [80]. Topotecan (TPT) is a DNA topoisomerase I inhibitor that has shown anti-cancer effects on gastric cancer and the results from its testing suggest that ASCT2 is an anti-cancer target for this compound, as it presented the same effects as ASCT2 knockdown [79]. Furthermore, the silencing of this protein with antisense RNA in hepatic adenocarcinoma cells did not appear to be attributed to glutamine or overall amino acid deprivation, thus suggesting a crucial role of ASCT2 in cell apoptosis beyond maintaining of amino acid homeostasis [81]. In fact, whereas both ASCT2 silencing and glutamine starvation promoted caspases-2 and -3 activity, there was no evidence for caspases-8 and -9 activities in glutamine-starved cells [82]. Moreover, both caspases-2 and -9 are upregulated in response to ASCT2 suppression and showed to be involved in the intrinsic pathway of apoptosis in response to oxidative stress, which in turn may result from the loss of glutathione (GSH), an antioxidant, due to the decrease of its precursors glutamine and cysteine, amino acids transported by ASCT2 (Figure 4) [82]. Knockdown or inhibition of this transporter has been associated with increased levels of ROS and decreased levels of GSH in gastrointestinal cell lines and with significant loss of mitochondrial potential in lung cancer cells [79,80,83]. The epidermal growth factor receptor (EGFR) is involved in downstream signal transduction of several pathways upon binding of a specific ligand, which may result in cell proliferation or evasion of apoptosis [84]. ASCT2 has been identified as an EGFR-associated protein that can be co-targeted by cetuximab, an EGFR antibody approved for metastatic human head and neck squamous cell carcinoma treatment, sensitizing the cancer cells to ROS-induced apoptosis through reduction of intracellular levels of glutamine and, consequently, of glutathione [85].

Dysregulation of LAT1 transporter has also been shown to affect caspase activity and, consequently, apoptosis [20]. Treatment of the KB human oral epidermoid carcinoma cells, Saos2 human osteogenic sarcoma cells and C6 rat glioma cells with BCH induced DNA fragmentation and increased TUNEL-positive cells [86]. Moreover, LAT1 inhibition led to the activation through proteolytic cleavage of caspase-3 and -7, which are synthesized as inactive proenzymes, in KB and C6 cells, inducing apoptotic cell death [86]. In Saos2 cells, this induction only depended on caspase-7 activation [86]. Similarly, BCH treatment also induced TUNEL-positive apoptotic cells from a human malignant glioma cell line [87]. Furthermore, this effect correlated with cleavage induction of both caspase-3 and its intrinsic substrate PARP [87].

### 2.2. Sustaining Proliferative Signaling

The ability to sustain chronic proliferation depends on the release of growth-promoting signals and cell cycle progression [6]. The phosphoinositide 3-kinase (PI3K)/Akt/mammalian target of rapamycin complex (mTORC) 1 pathway is critical for cell growth, integrating signals from a variety of factors, such as energy status and growth factors, and is highly supported by nutrients such as amino acids, particularly leucine, which is required for the activation of mTORC1 and is transported into the cell by LAT1 [3,111]. mTORC1 and mTORC2, the two known mTOR complexes, are inhibited during amino acid starvation, leading to a decrease in mRNA and protein biosynthesis resulting from alterations in the phosphorylation pattern of their downstream effectors, namely ribosomal protein S6 kinase beta-1 (S6K1) and eukaryotic translation initiation factor 4E-binding protein 1 (4E-BP1) [5]. mTORC1 activation occurs at the lysosomal membrane and its rate-limiting step is the uptake of l-glutamine via ASCT2 [5,112]. After 1–2 min, that amino acid leaves the cell, leading to the uptake of l-leucine by LAT1 and the rapid activation of S6K1 through mTORC1 [5].

The role of ASCT2 in tumor growth, namely its association with the inhibition of mTOR pathway, has been observed in breast cancer [113], melanoma [114], hepatoma [81], endometrial carcinoma [31], acute myeloid leukemia [115], gastric cancer [116], and prostate cancer [38], through either lentiviral transduction of a short hairpin RNA (shRNA) against ASCT2 or treatment with ASCT2 inhibitors (benzylserine or GPNA, for example). In fact, a study by Avissar et al. [88] showed that the PI3K pathway induced glutamine transport and ASCT2 expression in the human enterocytes cell surface. C-Myc is a well-characterized oncogene also involved in tumor cell growth [89]. It is a transcriptional factor frequently overexpressed in cancer and its levels have been correlated with ASCT2 and mTORC1 activation [89]. Additionally, the tumor suppressor retinoblastoma protein (Rb) is involved in cell cycle arrest by disabling the E2F family of cell cycle-promoting transcription factors and an inverse association with ASCT2 overexpression has been reported [90]. Furthermore, upregulation of SNAT1 and SNAT2, members of the transport System A and of the SLC38 family, was reported in ASCT2-deficient cells [91,92]. The substrate specificity of these transporters overlaps that of ASCT2 and they appear to explain the normal mTOR signaling observed in some cells lacking ASCT2 [91].

Leucine is a regulator of mTORC1, as previously mentioned, and also a major substrate for LAT1, which mediates its uptake in exchange for glutamine [5]. Pharmacological inhibition of LAT1, using the selective inhibitor JPH203, significantly reduced cell proliferation associated with mTORC1 pathway in six human cell lines without reducing CD98 expression [93]. In fact, Milkereit et al. [94] showed that LAT1-4F2hc is recruited to lysosomes by the lysosomal-associated transmembrane protein 4b (LAPTM4b), promoting leucine uptake into those organelles and mTORC1 activation. LAT1 inhibition has been associated with reduced cellular proliferation, often associated with reduced phosphorylation level of mTOR and its downstream effectors in several cancer models, including lung [52,95], colorectal [43] and breast cancer [96]. Similarly, LAT1 expression has been associated with increased tumor size and tumor cell growth rates [87,97]. On the other hand, Fan et al. [117] observed little impact of BCH treatment on ovarian cancer cells proliferation. Besides LAT1, this inhibitor is also able to block the activity of other leucine-accepting transporters and the authors conclude that LAT1-mediated amino acid transport alone might have limited impact on anchorage-dependent cancer cell proliferation in tumors [117]. Concerning other pathways, LAT1 inhibition by the same compound induced cell cycle arrest at G1 phase in oral cancer cells by inhibiting the cyclin D2-cyclin-dependent protein kinase 6 (CDK6) complex and increasing p27 expression, a CDK inhibitor [98]. The cyclin/CDK complexes play major roles in cell cycle events, as the binding between those molecules constitutes a target of checkpoint pathways that ensure cell cycle progression [99]. EGFR mutations have been found in lung cancer and they have been shown to have significant responses to EGFR tyrosine kinase inhibitors (TKIs), such as gebitinib and erlotinib [84]. Imai et al. [95] observed higher LAT1 expression in NSCLC samples without EGFR mutations, which might be associated with TKIs refractoriness and a poor prognosis in combination of LAT1 expression and EGFR wild-type.

Since LAT1 is an obligatory exchanger and that leucine uptake is dependent on the intracellular concentration of the exchange substrates, functional amino acid transporter-coupling between ASCT2 and LAT1 has been proposed [118]. In that model, ASCT2 drives glutamine uptake, which serves as an exchange substrate for the LAT1-mediated leucine transport to the intracellular environment, allowing the activation of the mTORC1 pathway and cellular growth, as previously mentioned [118]. A reciprocal regulatory connection has also been observed between ASCT2, LAT1 and mTOR in prostate cancer, hepatoma, lymphoma and leukemic cells, since glutamine starvation-induced LAT1-mediated transport of amino acids or expression and/or leucine deprivation induced the same effects on ASCT2 [3,38,67]. However, Cormerais et al. [119] did not observe a disruption in LAT1 or mTORC1 activity nor in leucine uptake following ASCT2 knockout in colon LS174T and lung A549 adenocarcinoma cells. Nevertheless, ASCT2 ablation decreased tumor growth, suggesting a LAT1-independent role in tumor proliferation [119]. Overall, despite their dominant role in amino acid transport and involvement in cell proliferation, ASCT2 and LAT1 have been classified as “harmonizers” instead of drivers of amino acid signaling and accumulation, since cells seem to adapt to the knockout of those transporters [120].

### 2.3. Activating Invasion and Metastasis

Local invasion and distant metastasis reflect the tumor progression to higher grades of malignancy, representing the major cause of morbidity and death in cancer patients, and usually represent alterations in cell-cell adhesion and the attachment to the extracellular matrix (ECM) [6].

There are few published studies reporting the association between ASCT2 and invasion and metastasis and the underlying mechanism remains elusive. Nevertheless, a higher expression of *SLC1A5* in gastric cancer tissues has been correlated with clinicopathological features such as local invasion and lymph node metastasis [33]. Furthermore, *SLC1A5* downregulation in vitro resulted in significant inhibition of gastric cells invasion and migration [33].

Similarly, LAT1 expression has been found to be significantly higher in metastatic sites compared to primary tumors in a variety of cancers [41,58,111,121]. In parallel, higher LAT1 expression has been associated with an increase in metastatic lesion diameter [122]. Blockade of this transporter efficiently inhibited anchorage-independent growth of ovarian cancer cell lines, suggesting an important role in proliferation under non-adhesive conditions as the ones involved in migration and metastasis [117]. Moreover, cholangiocarcinoma cells ability to migrate and invade was reduced by a downregulation of LAT1 expression through shRNA and it is suggested that it may involve 4F2hc-related signaling, since this molecule interacts with β1 integrin and forms a complex that takes part in Akt signaling activation, contributing to carcinogenesis [73]. Cells lacking LAT1 showed a reduction in cell migration due to a decrease in phosphorylation and consequent activation of ERK1/2 signaling pathway, regulated by the reorganization of the collagen matrix that is mediated by the α1β1 integrin [73]. Overexpressed LAT1 together with 4F2hc has been reported as necessary for metastasis in colon cancer patients [74]. Ding et al. [75] determined that the *SLC7A5* transcript stability was increased by genetic suppressor element 1 (GSE1) in a post-transcriptional manner independent from direct binding. GSE1 is positively associated with many clinicopathological features, namely lymph node metastasis, clinical stage, depth of invasion and histological grade and LAT1 appears to mediate its functions and to be positively regulated by GSE1 [75]. Wang et al. [47] identified LAT1 as a downstream target of the CRK-like (CRKL) protein, a promoter of gastric cancer. Depletion of CRKL in a gastric cancer cell line resulted in impairment of *SLC7A5* expression and suppression of cell motility [47].

### 2.4. Inducing Angiogenesis

Tumor cells with high proliferation rates require high levels of nutrients and oxygen, as well as an efficient ability to dispose of carbon dioxide and metabolic wastes [6]. These needs are addressed by the process of angiogenesis, which allows neovascularization associated with the tumor [6].

The expression level of both ASCT2 and LAT1 showed a significant correlation with CD147, an inductor of matrix metalloproteinases and tumor angiogenesis [76]. LAT1 expression correlated with that of vascular endothelial growth factor (VEGF), a marker of angiogenesis, in thymic carcinomas [77], colon [74] and lung cancers [63]. Furthermore, LAT1 has been detected in vascular endothelial and its expression level has been associated with glioma microvessel density [78].

### 2.5. Avoiding Immune Destruction

Immune surveillance recognizes and eliminates the majority of incipient cancer cells through the constant monitoring of tissues and cells by the immune system [6]. However, developing tumors appear to be able to evade immune recognition and thus avoid destruction [6]. In fact, an increase in tumor incidence has been observed in patients with deficiency of natural killer (NK) cells, CD8^+^ cytotoxic T lymphocytes (CTLs) and Th1 cells [6].

Interestingly, both ASCT2 and LAT1 have been reported as mediators of amino acid supply to activated T cells and important factors for T cell differentiation into Th1 and Th17 cells [7,68]. Moreover, ASCT2 transporter is required for mTORC1 signaling activation stimulated by T-cell receptor (TCR) and CD28, but not in other T cell activation pathways, making it particularly important in naïve T cells although dispensable in effector T cells, where it might be functionally redundant with other amino acid transporters [69]. LAT1 expression has been shown to be induced by CD3, which forms a complex with the TCR and allows its signaling, and CD28, a heterodimer that not only binds to integrin-β and mediates survival and growth through adhesive signals but is also involved in amino acid transport [68,70,71]. T cells need to achieve high levels of amino acid incorporation for normal immune responses, therefore requiring LAT1 to maintain their activated state [68]. In fact, T cells appear to use LAT1 to sustain the high demand for nutrients required for immune reactions in the same way cancer cells also prefer using this transporter to efficiently proliferate [68]. Despite ASCT2 importance in T cell activation, some studies have shown that, in ASCT2-knockout mice, this amino acid transporter is not required for T and B cell development, B cell proliferation and antibody production, suggesting that the immune system of cancer patients might tolerate the inhibition of this transporter [67,72].

### 2.6. Dysregulating Cellular Energetics

The uncontrollable proliferation rates that characterize cancer cells require adjustments in cell energy metabolism so that it can fuel the growth [6]. Glutamine is a non-essential amino acid that cells can synthesize de novo [123]. Nevertheless, it is one of the most important amino acids in the human body, being a source of both reduced nitrogen for biosynthetic reactions and of carbon for the mitochondrial tricarboxylic acid (TCA) cycle, also serving as a precursor to nucleotide and lipid synthesis [8,123,124]. Glutamine catabolism involves the conversion of glutamine to glutamate, a precursor of the TCA cycle intermediate α-ketoglutarate (α-KG), by cytosolic glutamine aminotransferases or mitochondrial glutaminases [123]. On the other hand, α-KG can be used to endogenously synthesize glutamine via the enzymes glutamate dehydrogenase and glutamine synthetase [125]. The need for glutamine is enhanced when cells undergo a high proliferation rate, with many tumors depending on glutaminolysis to fuel their energetic and metabolic necessities [5,14]. This process yields increased levels of downstream metabolites such as α-KG, pyruvate and lactate [5]. Additionally, the TCA cycle, where glutamine ends its fate, generates NADH and FADH_2_ that provide electrons for mitochondrial ATP generation [5,125]. Simultaneously, glutaminolysis has been considered a hallmark of cancer and its increase in cancer cells has been associated with an upregulation of some amino acid transporters, particularly ASCT2, whose targeting may lead to promising anti-cancer treatments [5,14].

Alteration of glutamine uptake has been reported as a result of the action of proto-oncogenes or tumor suppressors, similar to their action in stimulating cell proliferation as aforementioned [126]. C-Myc has been found to bind to promoter elements of the gene encoding ASCT2, resulting in higher levels of *SLC1A5* mRNA and enhanced glutamine uptake [100]. On the other hand, deletion of the tumor suppressors belonging to the Rb family alters glutamine metabolism by increasing the uptake of this amino acid through E2F transcription factor 3 (E2F3) binding to the *SLC1A5* promoter [90]. RNF5 is an E3 ubiquitin ligase associated with the endoplasmic reticulum involved in the regulation of protein stability and clearance, allowing the functioning of various cellular processes [101]. This protein has been found to play a role in ASCT2 degradation, also resulting in reduced glutamine uptake, induced by protein misfolding in response to endoplasmic reticulum stress [101]. There are also published studies on the influence of the tumor microenvironment on glutamine metabolism, contributing to tumor progression, involving cytokines and chemokines [102,127]. Interleukins (ILs) are secreted proteins that interact with specific receptors and allow communication between cells [128]. IL-4 is a Th2 cytokine that interacts with the IL-4 receptor (IL-4R) and has been reported as a regulator of high ASCT2 mRNA and protein expression, enhancing glutamine uptake in breast cancer cells [102]. IL-3, required for survival and proliferation of hematopoietic progenitor cells, binds to IL-3Rα, a glucose-dependent receptor, and signaling through this receptor induces ASCT2 [103]. Moreover, Jak inhibition repressed glutamine uptake, completely blocking ASCT2 induction, suggesting that IL-3 action occurs via the Jak/signal transducer and activator of transcription (STAT) pathway [103]. Ren et al. [104] have shown that neuroblastomas with amplification of the MYCN gene, which is associated with high levels of the N-Myc protein, rely on high amounts of glutamine to sustain cell viability, the TCA cycle and biosynthetic activities and that those high amounts are supported by ASCT2 activation. The activation of this transporter appeared to occur via activating transcription factor 4 (ATF4), a member of the cyclic AMP response element binding protein (CREB) family frequently induced in response to hypoxia, endoplasmic reticulum (ER) and oxidative stress and nutrient deprivation, in coordination with N-Myc [104]. There are also studies reporting the epigenetic modulation of tumor glutamine metabolism via ASCT2. In a study by Dong et al. [105], inhibition of the tumor suppressor microRNA-137 (miR-137) through methylation of its promoter by DNA methyltransferases (DNMTs) together with methyl-CpG-binding protein 2 (MeCP2) led to ASCT2 upregulation and, consequently, glutamine metabolism reactivation, identifying miR-137 as a regulator of glutaminolysis by targeting ASCT2 [105]. Interestingly, this miR has also been identified as a regulator of ferroptosis, an iron-dependent form of cell death genetically and morphologically different from apoptosis, by directly targeting ASCT2 in melanoma cells [106]. miR-137 suppressed ferroptosis, inhibiting the ability of melanoma cells to colonize in vitro and develop tumors in a mouse model [106]. Ferroptosis inhibition led to ASCT2 overexpression, which rescued the suppressing effect of miR-137 in this form of cell death [106].

Despite its high affinity for glutamine and the fact that *SLC1A5* knockout did decrease the uptake of this amino acid, blockade of ASCT2 in leukemic cells resulted in a much more pronounced effect on global cell metabolism which cannot be explained solely by glutamine transport [67]. Other amino acids play important roles in biosynthesis and intracellular redox homeostasis in cancer cells, such as cysteine, isoleucine, threonine and valine, and they have been found to significantly decrease in *SLC1A5*-knockout cells [67]. Furthermore, the pentose phosphate pathway, the glycolytic pathway, as well as the methylation cycle, had decreased rates in leukemic cells in the same study by Ni et al. [67].

Leucine plays important signaling roles, promoting protein synthesis through the activation of the mTOR pathway, enhancing mitochondrial biogenesis and augmenting fatty acid oxidation [129]. As previously mentioned, this amino acid can be transported into the cell by LAT1 [129]. Treatment of NSCLC cells with delta-tocotrienol (δT) inhibited both LAT1 and ASCT2 expression, resulting in a significant decrease in leucine concentration [107]. Furthermore, treatment of breast cancer cells with JPH203, a tyrosine analog and selective LAT1 inhibitor, limited the amount of leucine, and also tyrosine, that could maintain protein production or enter the TCA cycle, proving to be beneficial in combination with other mTOR inhibitors and/or endocrine therapies for breast cancer treatment [108].

## 3. ASCT2 and LAT1: Their Clinical Significance in Cancer

**Figure 5:**
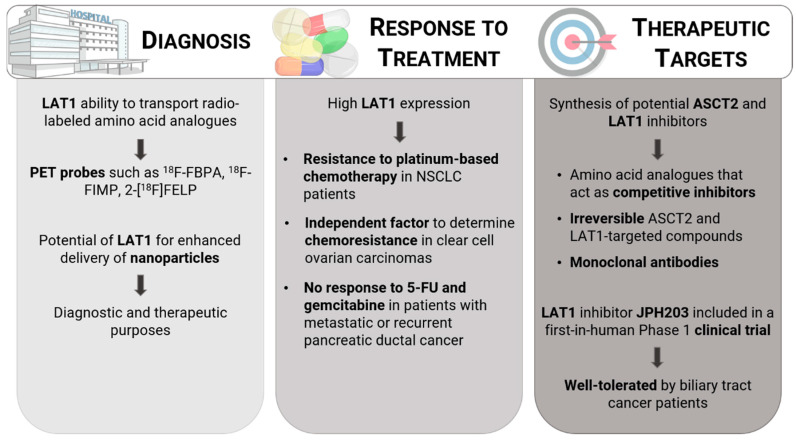
Clinical significance of ASCT2 and LAT1. LAT1 has been used in the clinic for diagnostic purposes due to its ability to transport PET probes and enhance nanoparticles delivery. Moreover, high LAT1 expression has been associated with chemoresistance in some types of cancer. ASCT2 and LAT1 have also been identified as therapeutic targets, with some studies reporting the successful synthesis of potential competitive or irreversible inhibitors of these transporters, as well as the use of monoclonal antibodies.

After acknowledging the potential role of the neutral amino acid transporters addressed here on the hallmarks of cancer, the focus of researchers and pharmaceuticals on ASCT2 and LAT1 clinical relevance is to be expected [130] (Figure 5). If these proteins are overexpressed in tumor cells, their blockade would likely lead to amino acid deprivation, impairing protein synthesis and, consequently, growth, while not affecting the biology of normal cells [131]. Moreover, drugs based on transporters are proposed to reduce possible side effects and enhance clinical efficacy [132].

### 3.1. ASCT2 and LAT1 as Prognostic Biomarkers in Cancer

The prognostic value of LAT1 in cancer has recently been the subject of two meta-analyses [133,134]. Those reviews reported associations between high LAT1 expression and poor overall, cancer-specific, disease-free and progression-free survival, as well as clinicopathological features such as stage, tumor size, lymphatic and vascular invasion, tumor differentiation, Ki-67, 4F2hc, CD34, p53 and ASCT2 [133,134]. LAT1, besides hepatocellular [39,48], lung [49,50,51,60,62,63,64,135,136], colorectal [44,137,138], renal [42,59], ovarian [40,139], breast [57], tongue [140] and pancreatic cancer [54,55], has also been identified as a prognostic marker for multiple myeloma [141], melanoma [142], cholangiocarcinoma [143], biliary tract [144,145], laryngeal [146], bladder [147], prostate [65,148,149], thymic [150] cancer and endometrioid carcinoma [46], even though the latter showed contradicting results.

Although with fewer published studies, ASCT2 has been identified as a prognostic factor in hepatocellular [34], lung [35], colorectal [151], gastric [33], renal [28], ovarian [36,152], breast [153], tongue [140] and pancreatic cancer [76]. Furthermore, genetic variants in the *SLC1A5* gene have also been implicated in hepatocellular carcinoma prognosis, with stage I patients carrying the rs2070246 TT genotype showing higher overall survival (OS) than carriers of CC genotype [154]. Concerning the latter, contradicting results had been previously reported by Watanabe et al. [45], who suggested a low significance of LAT1 as a prognostic marker since its expression had been low in poorly differentiated adenocarcinomas and correlated with neither proliferative activity nor the International Federation of Gynecology and Obstetrics stage.

Furthermore, co-expression of both transporters, ASCT2 and LAT1, has also been identified as an independent prognostic factor for lung adenocarcinoma [155] and surgically resected esophageal squamous cell carcinoma [156]. In fact, the combined expression of the transporters presented an overall poorer prognosis compared to the single-positive expression of ASCT2 or LAT1 in both models [155,156].

### 3.2. LAT1 as a Predictive Factor in Cancer Treatment

The relationship between the expression level of amino acid transporters, particularly of LAT1, and response to chemotherapy in cancer patients as been reported [53]. In NSCLC, LAT1 expression was significantly associated with resistance to platinum-based chemotherapy in patients with postoperative recurrence [53]. Higher expression LAT1 has also been identified as an independent factor for chemoresistance in ovarian clear cell carcinomas [139]. A similar role in chemotherapy resistance has been reported for LAT1 in patients with metastatic or recurrent pancreatic ductal adenocarcinoma after surgical resection, with all patients with high LAT1 expression being identified as non-responders to fluorouracil (5-FU) and gemcitabine [149].

### 3.3. ASCT2 and LAT1 Therapeutic Targets in Cancer

The research on the ASCT2 and LAT1 transporters as novel therapeutic targets for cancer therapy has been reported in the last five years (Table 2) [132,157,158,159]. The drug design of targets for these transporters usually follows an approach based on substrate analogues, which act as competitive inhibitors [159]. Several amino acid analogues have been reported to competitively bind to the ASCT2 substrate-binding site, impairing the amino acid transport, such as benzylserine [160], GPNA, the most potent compound of a series of glutamine analogues synthesized by Esslinger et al. [161], and V-9302, which selectively targets ASCT2 and represents the only inhibitor of this transporter that reached a preclinical phase [83,159], as well as other molecules based on sulfonamide/sulfonic acid ester scaffolds [162]. The major limitations of this type of inhibitors is the fact that (1) endogenous substrates can displace them from the binding pocket, impeding their effect; and (2) these transporters usually recognize more than one amino acid as substrate [159]. Thus, it is important to design compounds that are able to irreversibly target and block ASCT2 transport. l-phenylglycine and its analogues have been identified as non-substrate molecules that selectively and effectively inhibit the function of this transporter [163]. Furthermore, analogues of the amino acid proline, which is not a substrate for ASCT2, such as benzylproline-derived compounds have also been found as potential inhibitors [164,165]. Due to the strong reactivity of ASCT2 toward metals like mercury and its derivatives that form bonds with cysteine residues, the design of inhibitors that exploit this interaction could also play an important role in the irreversible inhibition of this transporter [14]. The use of monoclonal antibodies (MAbs) has also shown potential as an effective treatment for some cancers, namely in the suppression of glutamine-dependent growth of colorectal cancer cells (KM4008, KM4012 and KM4018) [166] and ASCT2-expressing gastric cancer (KM8094) [56,167].

Efforts have also been undertaken to synthesize potential LAT1 inhibitors due to the well-documented overexpression of this transporter in cancer [168]. Interestingly, GPNA, the widely used ASCT2 inhibitor, as already mentioned, has also been shown to impair the uptake of essential amino acids through other transporters, namely LAT1, lowering the cell content of these nutrients and potentially affecting mTORC1 activity [168]. Moreover, δT also inhibits both ASCT2 and LAT1 by impairing glutamine uptake, resulting in the induction of apoptosis and inhibition of cell proliferation due to mTOR pathway dysregulation [107]. BCH is a nonmetabolizable leucine analogue that has been shown to diminish growth in a variety of cancer cells [132,157,169]. Nevertheless, the concentration required to suppress cancer cell growth is extremely high and it constitutes a rather unspecific inhibitor of all the L-type amino acid transporters (LAT1-4) [157,158]. The anti-cancer potential of KYT-0353 or JHP203, a highly selective LAT1 inhibitor produced through synthetic chemistry and in vitro screening based on triiodothyronine (T3), has been reported in osteosarcoma [170], leukemic [171], oral cancer [172], gastric cancer [173], and colon cancer [173,174] cells, among others [157]. The thyroid hormone derivative 3-iodo-l-tyrosine has been identified as a potent LAT1 inhibitor through *cis*-inhibition and cell-proliferation experiments [175]. Other compounds have also been reported in recent years, including other inhibitors based on the T3 structure [176], with highlight to SKN103, and based on dithiazole and dithiazine compounds [177]. To the best of our knowledge, only the LAT1 inhibitor JPH203 is being tested in clinical trial setting [178]. In the in-human Phase 1 clinical trial study published by Okano et al. [178], clinical trial registration UMIN000016546, JPH203 was not only reported to have promising activity against biliary tract cancer, but was also well-tolerated in these patients. This compound is predominantly acetylated by N-acetyltransferase 2, which may exhibit distinct phenotypes according to its speed (rapid, intermediate, or slow) before it is excreted in bile [178]. Those phenotypes might be useful to predict JPH203 safety and efficacy [178].

### 3.4. LAT1 in Cancer Diagnosis and Drug Delivery

The potential of LAT1 for enhanced delivery of nanoparticles into the cancer cells for diagnostic and therapeutic purposes has been previously addressed [157,186,187]. Phenylalanine-coupled solid lipid nanoparticles (SLN) loaded with doxorubicin, a chemotherapy drug, have been shown to target LAT1 in the blood-brain barrier, as well as in brain cancer cells, where it is highly expressed, significantly enhancing the delivery of that drug and its anti-cancer activity [188]. The potential of this transporter as a nanoparticle target, either directed for chemotherapy or photothermal therapy via multi-branched gold nanoparticles, has also been reported for breast cancer treatment [189,190]. Furthermore, the uptake of liposomes modified with a LAT1-targeting polymer with thermoresponsive properties has also been enhanced in HeLa cells, suggesting the usefulness of this transporter as a target for drug delivery [191].

Interestingly, LAT1 has also the potential to be useful in cancer diagnosis due to its ability to transport radio-labeled (^18^F- or ^11^C-) amino acid analogues that function as positron emission tomography (PET) probes, allowing their visualization inside the body [158]. 2-^18^F-fluoro-2-deoxy-D-glucose ([^18^F]FDG) was one of the most commonly used PET probes, based on the fact that tumor cells consume high amounts of glucose, which led to false-positive results, especially in organs with higher uptake of glucose, like the brain [158]. To overcome this drawback, novel probes, including the amino acid analogues L-4-borono-2-^18^F-fluorophenylalanine (^18^F-FBPA) [192], (S)-2-amino-3-[3-(2-^18^F-fluoroethoxy)-4-iodophenyl]-2-methylpropanoic acid (^18^F-FIMP) [193] and 2-[^18^F]-2-fluoroethyl-L_-_phenylalanine (2-[^18^F]FELP), attracted attention [194].

## 4. Conclusions

The importance of amino acid transporters in cancer has been explored in the last two decades. However, there is a limited number of studies supporting the involvement of ASCT2 and LAT1 in tumor development for each cancer model. These transporters have been associated with a variety of hallmarks of cancer, such as resisting cell death, sustaining proliferative signaling, activating invasion and metastasis, inducing angiogenesis, avoiding immune destruction and dysregulating cellular energetics. Moreover, the clinical significance of these transporters has been explored in a variety of cancers. Due to their usefulness for cancer therapy and/or diagnosis, as well as their association with chemotherapy response, efforts should be undertaken to develop and test inhibitors that can be evaluated in clinical trials, such as the LAT1 inhibitor JHP203, hopefully enabling the improvement of cancer prognosis.

## Figures and Tables

**Table 1:** ASCT2 and LAT1 expression in cancer.

Authors ^1^ (Year) [Ref]	Cancer Model	Method	Transporter Expression in Cancer (*p* Value)	
Liu et al. (2015) [28]	Clear cell renal cell carcinoma	RT-qPCR	ASCT2 upregulation (*p* = 0.007)	
Huang et al. (2014) [29]	Colorectal cancer	IHC	ASCT2 upregulation	(*p* < 0.001)
		WB		(*p* < 0.05)
Schulte et al. (2017) [30]	Primary and metastatic colorectal cancer	IHC	ASCT2 upregulation in cancer (*p* < 0.001) and in metastases (*p* < 0.001) compared to normal tissue	
Marshall et al. (2017) [31]	Endometrial carcinoma	Risinger et al. [32] cohort analysis (Multiplex-qPCR)	ASCT2 upregulation in serous (*p* < 0.001) and endometrioid (*p* < 0.001) subtypes compared to normal tissue	
Lu et al. (2017) [33]	Gastric cancer	IHC	ASCT2 upregulation	(*p* < 0.0001)
		RT-qPCR		(*n* = 32, *p* = 0.0046; *n* = 45, *p* < 0.0001; *n* = 80, *p* = 0.0147)
Sun et al. (2016) [34]	Hepatocellular carcinoma	IHC	ASCT2 upregulation (*p* = 0.009)	
Shimizu et al. (2014) [35]	Non-small cell lung cancer	IHC	Higher ASCT2 expression in non-AC compared to AC (*p* = 0.019)	
Guo et al. (2018) [36]	Epithelial ovarian cancer	RT-qPCR and WB	ASCT2 upregulation (*p* < 0.001)	
Kaira et al. (2015) [37]	Pancreatic ductal carcinoma	IHC	ASCT2 upregulation (*p* < 0.001)	
Wang et al. (2015) [38]	Prostate cancer	TCGA cohort analysis	ASCT2 upregulation (*p* = 0.025) and higher ASCT2 expression in recurrent cancer compared to patients undergoing NHT (*p* < 0.001)	
Namikawa et al. (2015) [39]	Hepatocellular carcinoma	IHC	Higher ASCT2 expression compared to LAT1 (*p* < 0.001)	
Kaira et al. (2015) [40]	Ovarian cancer	IHC	Higher ASCT2 expression compared to LAT1 (*p* = 0.032), although no difference was found in epidermal ovarian cancer	
Papin-Michault et al. (2016) [41]	Brain metastasis	IHC	LAT1 upregulation (*p* < 0.001)	
Betsunoh et al. (2013) [42]	Clear cell renal cell carcinoma	RT-qPCR	LAT1 upregulation (*p* < 0.0001)	
Hayase et al. (2017) [43]	Colorectal cancer/Colonic adenoma	IHC	LAT1 upregulation compared to non-malignant tissue (NA) and colonic adenoma (*p* < 0.001)	
Sakata et al. (2020) [44]	Colorectal AC	WB	LAT1 upregulation compared to normal tissue (NA), with an increase in a sporadic adenoma-adenoma-carcinoma series (*p* < 0.0001)	
		IHC		
Watanabe et al. (2014) [45]	Endometrial endometrioid AC	IHC	LAT1 upregulation compared to atrophic endometrium (*p* < 0.01)	
Sato et al. (2020) [46]	Endometrial carcinoma	IHC	LAT1 upregulation in non-endometrioid compared to endometrioid carcinomas (*p* < 0.001)	
Wang et al. (2016) [47]	Gastric cancer	RT-qPCR	LAT1 upregulation (*p* < 0.01)	
Li et al. (2013) [48]	Hepatocellular carcinoma	RT-qPCR	LAT1 upregulation (*p* < 0.001)	
Kaira et al. (2008) [49]	Resectable stage I-III NSCLC	IHC	LAT1 upregulation (NA), higher LAT1 expression in SCC than AC (*p* < 0.001) and in non-AC than AC (*p* < 0.001)	
Imai et al. (2009) [50]	Stage I NSCLC	IHC	Higher LAT1 expression in SCC than AC (*p* < 0.001) and in non-AC than AC (*p* < 0.001)	
Takeuchi et al. (2010) [51]	NSCLC	RT-PCR	LAT1 upregulation (*p* < 0.05)	
Kaira et al. (2011) [52]	Resected NSCLC	IHC	Higher LAT1 expression in non-AC than in AC (*p* < 0.001)	
Kaira et al. (2011) [53]	NSCLC (recurrence after platinum-based chemotherapy)	IHC	Higher LAT1 expression in non-AC than in AC (*p* = 0.0022)	
Kaira et al. (2012) [54]	Pancreatic cancer	IHC	LAT1 upregulation (*p* < 0.001)	
Yanagisawa et al. (2012) [55]	Pancreatic AC	IHC	Higher LAT1 expression in IPMC compared to PDAC (*p* < 0.0001)	

AC: adenocarcinoma, ASCT2: alanine, serine, cysteine transporter 2, IHC: immunohistochemistry, IPMC: intraductal papillary mucinous carcinoma, LAT-1: L-type amino acid transporter 1, NHT: neoadjuvant hormone therapy, NSCLC: non-small cell lung cancer, PDAC: pancreatic ductal adenocarcinoma, RT-qPCR: reverse transcription real-time polymerase chain reaction, SCC: squamous cell carcinoma, TCGA: The Cancer Genome Atlas, WB: western blot. ^1^ Published studies with no statistical significant results were not included [27,56,57,58,59,60,61,62,63,64,65].

**Table 2:** ASCT2 and LAT1 pharmacological inhibitors.

Transporter	Inhibitor	Model	Reference
ASCT2	Benzylcysteine	Normal kidney	[160]
	Benzylproline derivatives	Normal kidney	[165]
	Benzylserine	Normal kidney	[160]
	δT	NSCLC	[107]
	GPNA	Rat glioma	[161]
	MAb KM8094	Gastric cancer	[56]
		Gastric cancer patient-derived xenograft mice	[167]
	MAb KM4008	Colorectal cancer	[166]
	MAb KM4012	Colorectal cancer	[166]
	MAb KM4018	Colorectal cancer	[166]
	Phenylglycine analogues	Wistar rat	[163]
		Normal kidney	[163]
	Sulfonamide/sulfonic acid ester scaffolds-based molecules	Normal kidney	[162]
	V-9302	Normal kidney	[83]
		Breast cancer	[83]
		Colorectal cancer	[83]
		Lung cancer	[83]
LAT1	BCH	Bladder cancer	[179]
		Breast cancer	[180]
		Cervical cancer	[86]
		Cholangiocarcinoma	[144]
		Endometrial cancer	[181]
		Esophageal cancer	[169]
		Glioma	[87]
		Head and neck	[182]
		Melanoma	[183]
		NSCLC	[95]
		Osteosarcoma	[86]
		Ovarian cancer	[58]
		Prostate cancer	[184,185]
		Rat glioma	[86]
	δT	NSCLC	[107]
	GPNA	Brain glioma	[168]
		Breast cancer	[168]
		Cervical cancer	[168]
		Hepatocellular carcinoma	[168]
		NSCLC	[168]
	JHP203	Colorectal cancer	[173,174]
		Gastric cancer	[173]
		Head and neck	[172]
		Leukemia	[171]
		Osteosarcoma	[170]
	SKN103	Cervical cancer	[176]
		NSCLC	[176]
		Pancreatic cancer	[176]

δT: delta-tocotrienol, ASCT2: alanine, serine, cysteine transporter 2, BCH: 2-aminobicyclo[2,2,1]-heptane-2-carboxylic acid, GPNA: L-γ-glutamyl-*p*-nitroanilide, LAT-1: L-type amino acid transporter 1, MAb: monoclonal antibody, NSCLC: non-small cell lung cancer.

## Data Availability

Not applicable.

## Abbreviations

4E-BP1	Eukaryotic translation initiation factor 4E-binding protein 1
5-FU	Fluorouracil
α-KG	α-Ketoglutarate
δT	δ-Tocotrienol
ASCT2	Alanine, serine, cysteine transporter 2
ATF4	Activating transcription factor 4
BAK	BCL-2 antagonist/killer
BAX	BCL-2-associated X protein
BBB	Blood-brain barrier
BCH	2-Aminobicyclo [2,2,1]heptane-2-carboxylic acid
BCL-2	B-cell lymphoma 2
CDK6	Cyclin-dependent protein kinase 6
CREB	Cyclic AMP response element binding protein
CRKL	CRK-like
CTL	Cytotoxic T lymphocytes
DNMT	Deoxyribonucleic acid methyltransferase
E2F3	E2F transcription factor 3
ECM	Extracellular matrix
EGFR	Epidermal growth factor receptor
ER	Endoplasmic reticulum
GPNA	L-γ-glutamyl-*p*-nitroanilide
GSE1	Genetic suppressor element 1
GSH	Glutathione
HAT	Heterodimeric amino acid transporter
IL	Interleukin
IL-R	Interleukin receptor
LAPTM4b	Lysosomal-associated transmembrane protein 4b
LAT1	L-type amino acid transporter 1
MAb	Monoclonal antibody
MeCP2	Methyl-cpg-binding protein 2
miR	Micro ribonucleic acid
MMP	Matrix metallopeptidase
mTORC	Mammalian target of rapamycin complex
NK	Natural killer
NSCLC	Non-small cell lung cancer
OS	Overall survival
PET	Positron emission tomography
PI-3K	Phosphoinositide 3-kinase
Rb	Retinoblastoma protein
ROS	Reactive oxygen species
S6K1	Ribosomal protein S6 kinase beta-1
shRNA	Short hairpin ribonucleic acid
siRNA	Small interfering ribonucleic acid
SLC	Solute carrier family
SLC1A5	Solute carrier family A1 member 5
SLC7A5	Solute carrier family A7 member 5
SLN	Solid lipid nanoparticles
SNAT	Sodium-coupled amino acid transporter
STAT	Signal transducer and activator of transcription
TCA	Tricarboxylic acid
TCR	T-cell receptor
TKI	Tyrosine kinase inhibitor
TM	Transmembrane segment
TNF	Tumor necrosis factor
TPT	Topotecan
VEGF	Vascular endothelial growth factor
